# Resisting majesty: *Apis cerana*, has lower antennal sensitivity and decreased attraction to queen mandibular pheromone than *Apis mellifera*

**DOI:** 10.1038/srep44640

**Published:** 2017-03-15

**Authors:** Shihao Dong, Ping Wen, Qi Zhang, Xinyu Li, Ken Tan, James Nieh

**Affiliations:** 1 Key Laboratory of Tropical Forest Ecology, Xishuangbanna Tropical Botanical Garden, Chinese Academy of Sciences, Kunming, China; 2 Eastern Bee Research Institute, Yunnan Agricultural University, Kunming, China; 3 Division of Biological Sciences, Section of Ecology, Behavior, and Evolution, University of California, San Diego, La Jolla, California, USA

## Abstract

In highly social bees, queen mandibular pheromone (QMP) is vital for colony life. Both *Apis cerana (Ac*) and *Apis mellifera (Am*) share an evolutionarily conserved set of QMP compounds: (*E*)-9-oxodec-2-enoic acid (9-ODA), (*E*)-9-hydroxydec-2-enoic acid (9-HDA), (*E*)-10-hydroxy-dec-2-enoic acid (10-HDA), 10-hydroxy-decanoic acid (10-HDAA), and methyl *p*–hydroxybenzoate (HOB) found at similar levels. However, evidence suggests there may be species-specific sensitivity differences to QMP compounds because *Ac* workers have higher levels of ovarian activation than *Am* workers. Using electroantennograms, we found species-specific sensitivity differences for a blend of the major QMP compounds and three individual compounds (9-HDA, 10-HDAA, and 10-HDA). As predicted, *Am* was more sensitive than *Ac* in all cases (1.3- to 2.7- fold higher responses). There were also species differences in worker retinue attraction to three compounds (9-HDA, HOB, and 10-HDA). In all significantly different cases, *Am* workers were 4.5- to 6.2-fold more strongly attracted than *Ac* workers were. Thus, *Ac* workers responded less strongly to QMP than *Ac* workers, and 9-HDA and 10-HDA consistently elicited stronger antennal and retinue formation responses.

Honey bee queens produce a pheromone, queen mandibular pheromone (QMP), which plays a central role in colony life and has multiple effects, depending upon the receivers and the context^1^,^2^,^3^. QMP can act as a sex pheromone and attract drones to virgin queens^3^,^4^. Within the colony, QMP signals the queen’s presence, inhibits worker ovarian development^5^,^6^, and maintains normal colony activity^7^. Interestingly, workers of the Asian honey bee, *Apis cerana (Ac*), have higher rates of ovarian activation than workers of the other *Apis* species, including *A. mellifera ligustica (Am*) and *A. florea (Af*)^8^,^9^. In colonies with a normal egg-laying queen (queenright colonies), about 5% of *Ac* workers have activated ovaries^10^,^11^,^12^. In comparison, 0.02% of *Am* workers and 0.01% of *Af* workers have activated ovaries^10^,^13^. Sakagami and Akahra (1958) similarly reported that about 10–20% of *Ac* workers contained mature eggs in their ovaries^14^. In contrast, about one *Am* worker in 1,000 contains visible eggs and only one worker in 10,000 contains a full-sized egg^8^.

QMP is also essential for creating the worker cluster (retinue) around the queen^3^,^10^,^13^. The attraction exerted by QMP reflects its central role and the importance of this retinue for grooming and feeding the queen and distributing QMP throughout the colony^7^. Aside from daily care, this retinue has implications for queen survival. For example, *Am* workers are more attracted to higher- as compared to lower-quality queens, and low queen attractiveness may contribute to queen replacement, a process in which workers play can play a role^15^. Most, QMP retinue studies have focused on *Am*^7^, but QMP also elicits retinue attraction in *Ac*^16^.

QMP is a blend of components^3^,^7^,^10^. In *Am*, six primary components have been identified (Table 1). The most abundant component, (*E*)-9-oxodec-2-enoic acid (9-ODA) was recognized more than 50 years ago^3^. Subsequently, (*E*)-9-hydroxydec-2-enoic acid (9-HDA), methyl *p*–hydroxybenzoate (HOB), 4-hydroxy-3-methoxyphenylethanol (HVA), (*E*)-10-hydroxy -dec-2-enoic acid (10-HDA) and 10-hydroxy-decanoic acid (10-HDAA) were identified^3^,^6^,^7^,^17^. These QMP compounds do not act in isolation. Using whole body extracts, Keeling *et al*. (2003) identified four additional compounds that function synergistically with QMP to attract a worker retinue^7^. All of the major compounds (9-ODA, 9-HDA, 10-HDA, 10-HDAA, and HOB) found in *Am* QMP are also found in *Ac* QMP (Table 1). However, *Ac* queens do not produce HVA^16^, and HVA does not increase retinue attraction of *Ac* workers when added to the other QMP compounds^5^.

These species differences could arise from multiple factors. However, a logical first step is to examine the sensory input (antennal olfactory sensitivity) and an immediate behavioral output, physical attraction and movement towards a component, which will, in turn, expose a worker to higher levels of that compound. Our goals were therefore to compare the antennal responses and retinue formation behaviors of *Ac* and *Am* workers to identical presentations of major-component QMP blends and individual QMP components. We focused on the major QMP components that are known to play a key role in retinue formation in both species^7^,^16^.

## Results

### *Am* had stronger antennal responses than *Ac*

**Figure 1:**
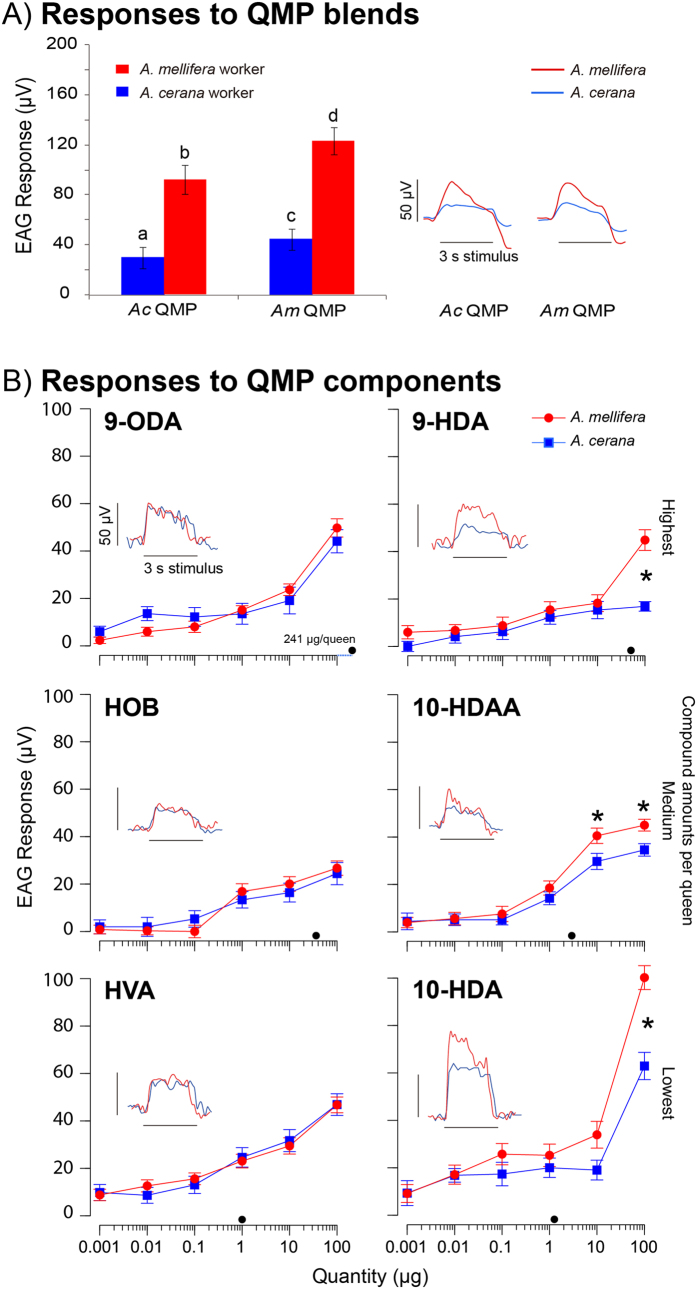
Antennal responses of *A. cerana (Ac*) and *A. mellifera (Am*) workers to synthetic QMP compounds. In all cases with significantly higher EAG responses, *Am* workers had stronger responses than *Ac* workers. Each plot shows the mean rectified EAG responses (response to blank solvent subtracted from the response to the test compound) with standard error bars. (**A**) Worker responses to the major QMP blends of *Ac* and *Am* queens. Significant differences are indicated with different letters (Tukey’s HSD test, *P* < 0.05). The EAG traces show typical responses to one queen equivalent of QMP blend. (**B**) Worker responses to individual compounds. The insets show a typical EAG response for a 100 μg dose of the test compound. Stars show significant differences based upon Least-Squares Means Contrast tests (*F*_1,86_ ≥ 6.32, *P* ≤ 0.014). Filled-in black circles on the x-axes show the mean quantity per queen, averaged for both species. Compounds are grouped into three rows, corresponding to the average amounts found in one queen equivalent of QMP (see Table 1).

To measure antennal sensitivities, we used electroantennograms (EAG), which are commonly used to measure olfactory stimulus sensitivity in honey bees^18^ and have been employed to measure worker responses to *Am* QMP^1^. In both species, the slope and shape of EAG antennal responses to different compounds were similar, and exhibited a fast recovery to baseline (Fig. 1). However, there were differences in the peak magnitudes of responses to different compounds.

We tested responses to major QMP blends that contained the most abundant components found in the QMP of each species (Table 1) and individual compounds. As predicted, *Am* had significantly stronger antennal responses to the major QMP blends and to some individual compounds than *Ac. Ac* never had significantly stronger responses than *Am* (Fig. 1). For the blends, there were significant effects of bee species (*F*_1,32_ = 51.95, *P* < 0.0001) and compound (*F*_1,34_ = 21.95, *P* < 0.0001), but no significant interaction of bee species*compound (*F*_1,34_ = 0.41, *P* = 0.53). Colony accounted for 17% of model variance. *Am* workers had a significantly higher EAG response to the *Am* QMP blend than to the *Ac* QMP blend (Tukey’s HSD test, *P* < 0.05). Similarly, *Ac* workers had a stronger response to the *Am* QMP blend than to their own *Ac* QMP blend (Tukey’s HSD test, *P* > 0.05). *Ac* workers had consistently lower EAG responses than *Am* workers (Fig. 1A).

We next focused on testing EAG responses to individual compounds. In the full model (Table 2), there were significant effects of compound, dose and multiple interactions (*P* ≤ 0.004) since species responded differently to different compounds and doses (Fig. 1B). We therefore next considered each compound separately, using a Bonferroni-corrected alpha = 0.025 (*k* = 2) for all tests conducted on these data.

For all compounds, response amplitudes increased with higher doses (9-ODA *F*_5,150_ = 88.63, *P* < 0.0001; 10-HDA *F*_5,165_ = 145.52, *P* < 0.0001; 9-HDA *F*_5,155_ = 46.30, *P* < 0.0001; 10-HDAA *F*_5,170_ = 170.66, *P* < 0.0001; HOB *F*_5,170_ = 51.47, *P* < 0.0001; HVA *F*_5,180_ = 105.19, *P* < 0.0001). There was no significant effect of species overall for any compound (*P* ≥ 0.10). Colony accounted for < 1 to 20% of model variances (9-ODA < 1%; 10-HDA 20%; 9-HDA < 1%; 10-HDAA < 1%; HOB < 1%, HVA 4%). The interaction species*dose was not significant (*P* ≥ 0.49) for 9-ODA, HOB or HVA. However, for the other compounds, there were significant effects of dose and the interaction species*dose.

Specifically, *Am* had higher responses than *Ac* to the higher doses of 9-HDA, 10-HDAA, and 10-HDA (interaction effects: 10-HDA *F*_5,150_ = 88.63, *P* < 0.0001; 9-HDA *F*_5,155_ = 10.90, *P* < 0.0001; 10-HDAA *F*_5,170_ = 4.09, *P* = 0.002). *Am* had significantly higher responses than *Ac* to larger doses, particularly at 100 μg (Least-Squares Means Contrast tests, *F*_1,86_ ≥ 6.32, *P* ≤ 0.014, Fig. 1B). *Ac* did not have a higher response than *Am* to any tested compounds.

### *Am* was more strongly attracted to individual QMP compounds than *Ac*

**Figure 2:**
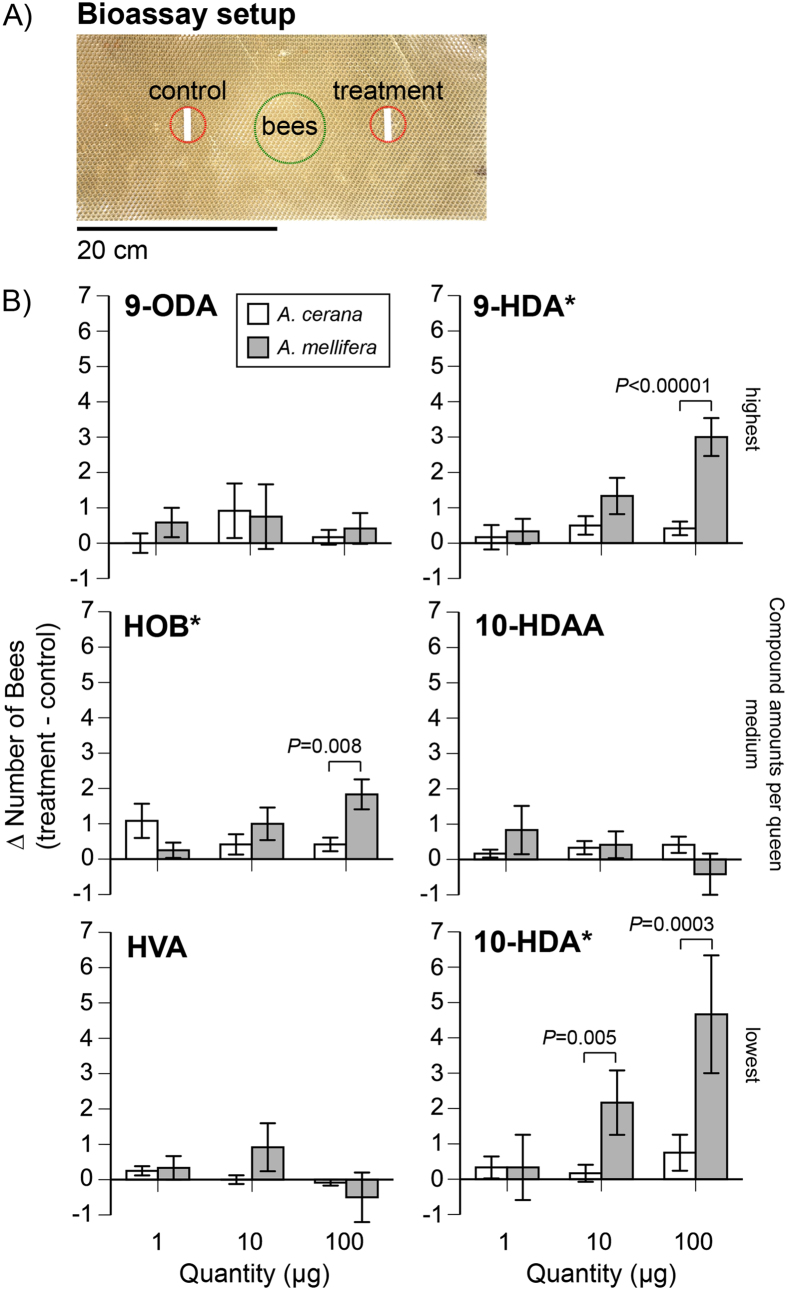
Bioassay of worker attraction (retinue formation) to synthetic queen mandibular pheromone (QMP) components. In all cases with significantly higher attraction, *A. mellifera (Am*) workers were more attracted than *A. cerana (Ac*) workers. (**A**) Photo of the wax comb foundation and filter papers with odor treatments, with dashed red circles showing the areas within which bees were counted. Bees were initially placed within the center zone circumscribed in green. (**B**) The mean per-trial difference in the number of bees that were attracted to the treatment as compared to the control is shown. Significantly more *Am than Ac* workers were attracted to higher quantities of 9-HDA, HOB, and 10-HDA (*). Means, standard errors, and significant contrast tests are shown. Compounds are grouped into three rows, corresponding to the average amounts found in one queen equivalent of QMP (see Table 1).

We measured the attraction of individual QMP compounds with a retinue bioassay. We counted the number of *Am* and *Ac* workers that moved across a comb towards the test compounds (Fig. 2). Three compounds (9-HDA, HOB, and 10-HDA) attracted significantly more *Am* than *Ac* workers. *Ac* was never significantly more attracted than *Am* (Fig. 2).

For 9-HDA, there was a significant effect of dose (Likelihood-Ratio *χ*^2^_1_ = 10.05, *P* = 0.007), no effect of species (L-R *χ*^2^_1_ = 2.33, *P* = 0.13), but a significant interaction (L-R *χ*^2^_2_ = 12.08, *P* = 0.002) because significantly more *Am* than *Ac* workers were attracted to the 100 μg dose (L-R *χ*^2^_1_ = 19.30, *P* < 0.00001).

For HOB, there were significant effects of dose (L-R *χ*^2^_2_ = 1.41, *P* = 0.50), species (L-R *χ*^2^_1_ = 4.08, *P* = 0.04), and the interaction dose*species (L-R *χ*^2^_2_ = 11.33, *P* = 0.004) because significantly more *Am* than *Ac* workers were attracted to the 100 μg dose (L-R *χ*^2^_1_ = 6.95, *P* = 0.008).

Higher doses of 10-HDA attracted more bees: there was a significant effect of dose (L-R *χ*^2^_2_ = 8.36, *P* = 0.015) but no significant effect of species (L-R *χ*^2^_1_ = 2.33, *P* = 0.13) or the interaction dose*species. (L-R *χ*^2^_2_ = 4.01, *P* = 0.13). However, graphical inspection of the data suggested the following contrast tests: significantly more *Am* than *Ac* workers were attracted to the 100 μg dose (L-R *χ*^2^_1_ = 12.81, *P* = 0.0003) and the 10 μg dose (L-R *χ*^2^_1_ = 7.78, *P* = 0.005).

For the remaining compounds, (10-HDAA, 9-ODA, and HVA) there were no effects of species (L-R *χ*^2^_1_ ≤ 3.42, *P* ≥ 0.06), dose (L-R *χ*^2^_2_ ≤ 3.42, *P* ≥ 0.94) or the interaction species*dose (L-R *χ*^2^_1_ ≤ 1.23, *P* ≥ 0.54), with one exception. There was a significant effect of 9-ODA dose (L-R *χ*^2^_2_ = 7.99, *P* = 0.02).

## Discussion

Two main results emerge from these experiments. First, *Am* workers consistently exhibited higher antennal sensitivity than *Ac* to the main components of QMP and the QMP blends. *Am* workers responded more strongly to the QMP blend of their own species, and *Ac* showed uniformly lower antennal responses. *Ac* did show a slightly higher response to the *Am* as compared to the *Ac* QMP blend, perhaps because the *Am* blend contained HVA but the *Ac* blend did not. HVA is not found in Ac QMP, but *Ac* workers show antennal responses to this compound. Second, *Am* also consistently showed a stronger retinue attraction than *Ac* to these QMP components. Such retinue attraction is important because it plays a major role in colony life, mediating care of the queen and helping to disperse QMP, which is a primer and a releaser of multiple important colony activities^7^.

In our individual compound tests, *Ac* workers never had higher antennal responses than *Am* to any compounds, and 9-HDA, 10-HDA, and 10-HDAA elicited higher amplitude antennal signals from *Am* than *Ac* workers (Fig. 1). The retinue bioassay likewise showed that only *Am* was more attracted to individual compounds (9-HDA,10-HDA, and HOB) than *Ac* (Fig. 2). The match between antennal sensitivity and behavioral attraction was not exact. Both 9-HDA and 10-HDA elicited strong antennal responses and attracted more workers. However, 10-HDAA elicited a stronger *Am* antennal response, but did not increase worker attraction. Likewise, HOB increased *Am* worker attraction but did not result in a higher antennal response. These differences likely arise from the role that higher order neural processing plays in worker attraction to QMP. However, the overall results of the retinue bioassay and the EAG measurements matched: *Am* consistently showed stronger responses than *Ac*.

It is possible that *Ac* and *Am* worker responses may depend upon the full blend of QMP compounds, including those found only in trace quantities. However, our goal was to provide comparative data by testing bees with identical doses of the same major compounds. Moreover, our *Ac* QMP blend contained the key compounds (9-ODA, 9-HDA, and HOB) that Plettner *et al*.^16^ found were sufficient to elicit a full *Ac* worker retinue response^16^.

What could cause these antennal sensitivity differences? They may have arisen from saturation differences in antennal responses. However, our major QMP blend tests (Fig. 1A) showed significant species differences at the level of one queen-equivalent. At this biologically relevant level, *Am* workers consistently had 1.8-fold higher antennal responses than *Ac* workers. Bees may also have perceived the compounds presented in isolation differently from a full QMP blend. Our results do not support this interpretation because the responses to the blends (Fig. 1A) are similar in amplitude to the sum of responses to compounds individually presented (Fig. 1B).

*Ac* antennae may be less sensitive to some QMP compounds (9-HDA, 10-HDAA, and 10-HDA) than *Am* antennae. In honey bees, differences in EAG antennal responses are associated with multiple morphological and electrophysiological properties of antennae^19^. The size and surface area of the antennae in *Ac* workers and *Am* workers appear to be identical, but the distributions of sensory hairs on the antennae are significantly different^20^. Four classes of olfactory sensilla (placodea, trichodeum types A and B, basiconica) are significantly more abundant on *Ac* than on *Am* worker antennae^20^. However, *Am* workers have a greater abundance of other sensilla, such as sensilla campaniformia, s. coeloconica, s. ampullaca, and s. chaetica, than *Ac* workers^20^. Insect sensilla abundance may affect antennal olfactory sensitivity^21^,^22^, although more studies are required to demonstrate this in honey bees. The number of different sensory neurons per sensilla also influences sensitivity, and the EAG response is a measure of summed responses of all chemosensory neurons in all antennal sensilla^23^.

Odorant receptors may differ between *Am* and *Ac*. Major groups of chemoreceptor genes include odorant receptors (Or’s)^24^. In *Am*, the number of odorant receptors has been estimated at 160–170, which is approximately equal to the number of glomeruli in the *Am* antennal lobe^24^,^25^. The *Am* receptor, Or11, has been functionally characterized as responding to 9-ODA^26^. In contrast, markedly fewer odorant receptors (119) have been characterized in the *Ac* genome^27^. *Am* may therefore have a better ability to detect odors than *Ac* in a variety of contexts. Finally, neuromodulators such as serotonin, octopamine and dopamine can alter the sensitivity of invertebrate chemosensory neurons^28^,^29^ and higher level processing should play a role. The evidently greater antennal sensitivity of *Am* as compared to *Ac* workers may derive from multiple factors.

Because QMP is distributed throughout a colony, average colony sizes are also relevant. *Ac* colonies contain fewer workers than *Am* colonies^30^. In our experiments, average *Ac* and *Am* colonies consisted of approximately 15,000 and 20,000 bees, respectively. Given that the QMP of healthy, fertilized queens of each species contains, on average, the same amount of 9-ODA, 10-HDA, and HOB (Table 1), one might expect that *Ac* workers would be exposed, per bee, to higher levels of QMP than *Am* workers. However, even at higher levels QMP component doses, *Am* workers consistently exhibited stronger antennal responses (Fig. 1) and greater attraction (Fig. 2) than *Ac* workers.

The greater attraction of *Am* as compared to *Ac* workers towards QMP blends and their components may have implications beyond retinue formation and suggest future studies. In both species, laying workers have higher amounts of QMP components than non-laying workers^31^. *Ac* workers also seem slightly more tolerant of worker-laid eggs as compared to *Am* workers^12^. Thus, reduced *Ac* attraction to QMP compounds and blends, as compared to *Am*, may reflect an increased tolerance of workers with developed ovaries. It is also possible that decreased *Ac* worker sensitivity to QMP could partially account for the higher ovarian activation levels seen in *Ac* workers in queen-right colonies. However, testing these hypotheses will require a different set of experiments that examine worker ovarian development and the effects of long-term exposure to major QMP components.

What are the evolutionary reasons behind these differences? A review of social insect pheromones suggests that social insect queen pheromones have evolved to provide an honest indication of queen quality rather than as coercive agents that chemically sterilize workers^16^. Tan *et al*.^17^ provided evidence for such honest QMP pheromone signaling in *Ac*^31^. Given this, we hypothesize that the higher levels of ovarian development in *Ac* as compared to *Am* workers reflects a beneficial adaptation that facilitates colony fitness. For example, queenlessness may occur more often in *Ac* than in *Am* because swarming and absconding are more common in *Ac* than in *Am*^32^. However, if this is an honest queen-worker signal, *Ac* queens could simply have evolved a lower level of QMP compounds rather than workers evolving a higher response threshold. Because both *Ac* and *Am* have similar levels of most QMP compounds (Table 1), we therefore wonder if other constraints, such as worker evaluation of and regulation of replacement queens^33^, are at play.

## Materials and Methods

### Study site and colonies

The experiments were conducted from May 2015 to August 2016, at Yunnan Agricultural University, Kunming, China. Three queenright *Ac* colonies and three *Am* colonies were kept in standard Langstroth hives. Each hive consisted of four frames covered with adult workers, two frames of brood and two frames of honey and pollen.

### Exp. 1: EAG

Over a wide variety of compounds, including 9-ODA, the electrophysiological responses of *Am* olfactory antennal cells increases up to 4 days of age after adult emergence and then remains fairly constant for the rest of adult life^34^. For example, Pham-Delegue *et al*.(1993) tested the EAG response of *Am* workers to *Am* queen-head extracts or to synthetic QMP (9-ODA, 9-HDA, HOB, and HVA in natural proportions) and found no significant differences in the magnitude of EAG responses for adult bees over the range of 2–21 days of age^35^. Similarly, Allan *et al*.(1987) tested the EAG responses of *Am* workers of different ages (1–60 days of adult age) to two components of *Am* QMP (9-HDA and 9-ODA) and showed that EAG responses increased in magnitude with age, but were roughly similar for bees between 6–40 days, particularly for 9-HDA^36^. Our approach assumes that, in *Am* and *Ac* workers of similar age, the same EAG response indicates a similar sensitivity. This assumption may not be correct, but we believe that this is a reasonable initial approach given that we have no *a priori* expectation of differences or the direction (higher or lower sensitivity) of any putative differences.

We used plastic boxes to carefully capture adult workers (returning foragers) of both species as they returned to their nest entrances. Based upon the age of first foraging in these species, these bees were likely more than 22 days of adult age. We chose foraging-age bees because one of the few other comparative studies on QMP in *Am* and different Asian honey bee species, including *Ac*, also used bees of foraging age^37^.

We tested worker EAG responses to a blend of the major components found in the QMP in each species. We used the average amount of each compound per species (Table 1). The major-component *Ac* QMP blend therefore contained 243 μg 9-ODA, 33 μg 9-HDA, 31 μg HOB, 1.3 μg 10-HDA, and 0.9 μg 10-HDAA, and the *Am* QMP blend contained 237 μg 9-ODA, 67 μg 9-HDA, 26 μg HOB, 1.2 μg 10-HDA, 4 μg 10-HDAA, and 2 μg HVA^13^,^37^,^38^. Thus, the *Am* QMP blend contained HVA, which is not found in the QMP of *Ac* (Table 1). Each blend was diluted in dichloromethane (Aladdin, CN), which is highly volatile and elicits a minimal EAG response^39^, and presented at a quantity equivalent to one queen. To compensate for individual variation, each bee was exposed to three treatments: the blank control (dichloromethane), the *Ac* QMP blend, and the *Am* QMP blend. We used workers from three *Ac* colonies (*n* = 18 different bees: 6 bees from each colony) and three *Am* colonies (*n* = 18 different bees: 6 bees from each colony).

We also measured EAG responses to the individual QMP components that we used in our blends. The mandibular glands of a mated, egg-laying queen contain approximately 200 μg 9-ODA, which comprises about 60% of the total secretion in *Ac* and *Am* queens^13^,^37^. We therefore used doses ranging from 1 ng to 100 μg. Each compound was diluted in dichloromethane and presented as the following concentration series: 0 ng/μl (solvent control), 1 ng/μl, 10 ng/μl, 100 ng/μl, 1000 ng/μl, 10,000 ng/μl, and 100,000 ng/μl. Each sample was applied to a filter paper strip. The paper strip was then placed inside the odor pipette after the solvent was evaporated for 10 s. This should not have resulted in appreciable evaporation of the test compounds because all tested QMP components have vapor pressures below 0.0001 kPa. We used multiple bees from three *Ac* colonies (*n* = 106 different bees: 35, 35 and 36 from colonies 1, 2, and 3 respectively) and three *Am* colonies (*n* = 103 different bees: 34, 34, and 35 from colonies 1, 2, and 3 respectively).

To record the antennal response, we gently removed one worker at a time from the box in which it was captured, cut off a randomly-chosen antennae (left or right), and placed it between glass electrodes filled with honeybee Ringer’s solution^39^. Blends were presented in the order described above. Individual compounds were tested in an ascending concentration series on each antennal preparation. In the QMP blend tests, we tested each antenna with both blends. However, in the individual compound tests, we only tested one compound type per antenna. We waited for 30 s between stimulations to provide sufficient recovery time and then provided a 3 s stimulus. We measured the baseline-peak amplitude (mV)^39^.

The EAG recording system was the same as described in Wang *et al*.^39^. The antennal preparation was placed 1 cm away from the outlet of a odor pipette (1 cm inner diameter, 15 cm long) that provided the test odor by combining a clean and wet continuous air flow (15 ml/s, 90% relative humidity) and a pre-filtered and wet pulsed air flow (5 ml/s, 90% relative humidity). The test odor stimulus was presented for 3 s. All measurements were conducted at 25 **°**C. To record the antennal responses, a modified EAG amplifier fed the amplified (21X) and filtered EAG signal into an HP34465A Digital Multi Meter (Agilent, USA) and BenchVue software (Keysight, USA) running on a PC for signal recording.

For the chemical standards, commercially available HOB, HVA, and 10-HDAA were obtained from the Aladdin Reagent Database Inc. (Shanghai, China). Isomerically pure 9-ODA and 10-HDA were synthesized using the Doebner–Knoevenagel condensation method^40^. The 9-HDA was synthesized by selective reduction of the keto group of 9-ODA with NaBH_4_^41^.

### Exp. 2: Behavioral attraction (retinue formation)

We conducted a retinue bioassay to test the attractiveness of six major QMP components. We individually tested 9-ODA, 9-HDA, HOB, 10-HDAA, 10-HDA, and HVA, each at a quantity which was an average of the amount found in queens of both species (see Table 1).

For each trial, we collected 30 foragers (see Exp. 1 methods) from the entrance of the focal colony. We used CO_2_ applied for 5 s to briefly anesthetize the bees, and then placed them in the center (4 cm diameter circle) of a processed beeswax comb foundation (41 cm × 19.5 cm) inside a box. Prior studies with *Am* workers used a similar approach and counted the number of workers entering an elliptical space around the test lure^16^,^42^.

Centered and separated by 20 cm apart, we fastened two clean pieces of filter paper (each 0.8 cm × 2 cm) with insect pins (Fig. 2A). One paper was the solvent control (1 μl of dichloromethane) and the other was the treatment compound at different doses (1 μg, 10 μg, or 100 μg in 1 μl of dichloromethane). We then counted the number of bees entering a circle circumscribing each of the paper strips over a 5 min trial (Fig. 2A). Based upon preliminary trials run for 20 min, we found that maximal choice was achieved within 5 min. We used new comb foundation, filter papers, and pins for each trial. Control and treatment positions were alternated between trials to avoid potential side bias. Each trial tested one compound at one dose and used a different set of workers. We conducted four replicates of each condition with each colony and used three colonies of *Ac* and three colonies of *Am*.

The beeswax foundation was commercially purchased and was produced from the wax of *Am* colonies melted at a minimum of 45 °C and maintained in a liquid state for an extended duration. Before use, this foundation had also been sitting at room temperature (21 °C) for over one year. Because of this processing, high levels of species- and colony-specific volatiles were likely not retained. This foundation therefore provided a neutral, yet more natural base upon which we could conduct our bioassay. Moreover, any remaining species-specific volatiles would have been dispersed throughout the wax foundation and therefore should not have affected attraction to the test compound as compared to the solvent control. Finally, for half of the test compounds (9-ODA, 10-HDAA, and HVA), *Am* showed the same level of attraction as *Ac*, suggesting that the wax foundation did not bias our results in favor of *Am*.

### Statistics

Because bees exhibited variance in their individual baseline response to control exposure (0 μg), we calculated a rectified (corrected) response, obtained by subtracting the control response (CR) from all other responses. We used JMP Pro v12.0.1 for all tests. All models met parametric assumptions, as determined by analyses of residuals.

We first used Repeated Measures Analysis of Variance (ANOVA, REML algorithm) to analyze the effects of compound, dose, species, and all interactions on the log-transformed rectified response. In this model, colony and bee ID were random effects, and all other effects were fixed. Each bee was exposed to different doses of only one compound type, and thus we nested bee identity within compound. Because there were significant interactions between species and compound and between species and dose, we then analyzed each species separately to explore these effects in detail. We used limited post-hoc Least-Squares (L-S) Means Contrast tests to make comparisons between the responses of differences. We applied a Sequential Bonferroni correction (*k* = 2) and alpha = 0.025 to these data and reported non-significant results as *NS*.

To analyze the comb bioassay results, we calculated the difference between the number of bees attracted to the treatment filter paper as compared to the blank control (∆ number of bees). We first tested for potential colony effects by running a General Linear Model (GLM, Poisson distribution, Identity link, Maximum Likelihood Estimation, Overdispersion corrected) for each species with colony as a fixed effect. Given that we found no significant colony effects (Likelihood-Ratio *χ*^2^_2_ ≤ 3.50, *P* ≥ 0.17), we then pooled the colony data for each species and ran the same GLM model with fixed effects for dose, species, and the interaction dose*species. Based upon data inspection, we used a limited number of Likelihood Ratio (L-R) Chi-square contrast tests to compare the attraction between the two species.

## Additional Information

**How to cite this article**: Dong, S. *et al*. Resisting majesty: *Apis cerana*, has lower antennal sensitivity and decreased attraction to queen mandibular pheromone than *Apis mellifera. Sci. Rep.* **7**, 44640; doi: 10.1038/srep44640 (2017).

**Publisher's note:** Springer Nature remains neutral with regard to jurisdictional claims in published maps and institutional affiliations.

## Figures and Tables

**Table 1:** QMP components of mated *A. cerana (Ac)* and mated *A. mellifera (Am)*, egg-laying queens (mean ± standard error).

Component	*Ac* (μg)	*Am* (μg)	Mean of *Ac* & *Am* (μg)	*Ac/Am* (mean ratio)	*P*
9-ODA	243.10 ± 28.0	237.95 ± 28.0	240.53	1.0	0.90
9-HDA	33.44 ± 18.9	67.10 ± 18.9	50.27	0.5	0.24
HOB	30.79 ± 10.31	25.67 ± 10.31	28.23	1.2	0.73
10-HDAA	0.91 ± 1.34	4.14 ± 1.34	2.53	0.2	0.12
10-HDA	1.3 ± 0.05	1.2 ± 0.05	1.25	1.1	0.12
HVA	0.0	2.0	1	0.0	—

Data is from Tan *et al*.^38^ with the exception of HVA (data from Slessor *et al*.^13^. Per compound, the mean of both species is shown because these quantities were used for the comb bioassay experiment. *P*-values are from Univariate ANOVA tests reported in Tan *et al*. (2009) and show that the compounds are found at similar levels in both queens of both species^38^. For HVA, it is not possible to compare levels statistically because only a single data point is available for *Am*.

**Table 2:** Results of Repeated-Measures ANOVA testing the EAG responses of bees from both species: *A. mellifera (Am*) and *A. cerana (Ac*).

Effect	*F*_*Degrees of Freedom*_	*F*-ratio	*P*-value
Compound	*F*_5,251_	4.59	0.0005
Species	*F*_1,1088_	0.004	0.95
Dose (μg)	*F*_5,1086_	108.22	<0.0001
Compound*Species	*F*_5,1088_	20.46	<0.0001
Compound*Dose	*F*_25,1086_	4.30	<0.0001
Species*Dose	*F*_5,1086_	3.54	0.0035
Species*Dose*Compound	*F*_25,1086_	0.81	0.74

Colony accounted for 0.2% of model variance. We used a Bonferroni-corrected alpha = 0.025.
