# Corrigendum: Resisting majesty: *Apis cerana*, has lower antennal sensitivity and decreased attraction to queen mandibular pheromone than *Apis mellifera*

**DOI:** 10.1038/srep46872

**Published:** 2017-06-30

**Authors:** Shihao Dong, Ping Wen, Qi Zhang, Xinyu Li, Ken Tan, James Nieh

Scientific Reports 7: Article number: 4464010.1038/srep44640; published online: 03 15 2017; updated: 06 30 2017

The original version of this Article contained a typographical error in the Abstract.

“Thus, *Ac* workers responded less strongly to QMP than *Ac* workers, and 9-HDA and 10-HDA consistently elicited stronger antennal and retinue formation responses”.

now reads:

“Thus, *Ac* workers responded less strongly to QMP than *Am* workers, and 9-HDA and 10-HDA consistently elicited stronger antennal and retinue formation responses”.

This has now been corrected in the PDF and HTML versions of the Article.

